# Sodium alginate hydrogel loaded with upconversion nanoparticles and magnesium ions enhances bone regeneration and photodynamic tumor therapy

**DOI:** 10.3389/fphar.2025.1543678

**Published:** 2025-03-10

**Authors:** Siyu Song, Xirao Sun, Yanfu Wang, Meng Wang, Zheng Shi, Danfang Sun, Dan Li, Jianduo Chen, Chengyue Wang

**Affiliations:** ^1^ Jinzhou Medical University School of Stomatology, Jinzhou, China; ^2^ Stomatology Hospital Affiliated of Jinzhou Medical University, Jinzhou, China; ^3^ School and Hospital of Stomatology, China Medical University, Shenyang, China; ^4^ Collaborative Innovation Center for Health Promotion of Children and Adolescents of Jinzhou Medical University, Jinzhou, China

**Keywords:** hydrogel, bone regeneration, tumor inhibition, UCNPs, PDT

## Abstract

**Introduction:**

Oral squamous cell carcinoma (OSCC) usually invades the jawbone over the course of the disease. Hence, it is necessary to consider the treatment of the tumor and repair of the jawbone, and the treatment process is very complicated. However, conventional therapy for OSCC mainly emphasizes tumor removal, which often fails to address the repair of jawbone defects and destroys residual tumor cells after treatment.

**Methods:**

In this study, we designed a composite hydrogel platform (SUMg) of sodium alginate loaded upconversion nanoparticles (UCNP) and magnesium particles (MPs), in which UCNP was coated with folic acid and chlorin e6 to prepare UFC. The physicochemical and biological properties of the prepared SUMg, including swelling test, element mapping, cell behaviors, *in vivo* osteogenic bioactivity and antitumor effect, were comprehensively evaluated.

**Results:**

*In vitro*, SUMg exhibited high cytocompatibility and promoted cell viability, proliferation, spreading, and osteogenesis owing to the incorporation of MPs, with SU10 Mg having the most significant effect. Under 980 nm laser irradiation, UFC induced a photodynamic therapy effect and destroyed surviving tumor cells. *In vivo* experiments further confirmed that SU10 Mg could promote bone regeneration, and under 980 nm near-infrared irradiation, the UFC produced local reactive oxygen species in the tumor within 5 min.

**Discussion:**

This work demonstrated the potential of SUMg in bone regeneration and inhibition of tumor growth, providing valuable insight for OSCC treatment.

## Graphical Abstract

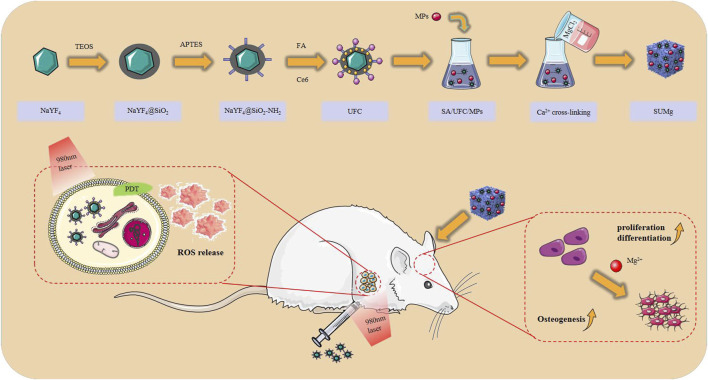

## 1 Introduction

Oral squamous cell carcinoma (OSCC) is the most prevalent malignant tumor of the maxillofacial region (He et al., 2020; Zhang et al., 2020). According to the latest global cancer statistics report, OSCC accounts for more than 370,000 new cancers and 170,000 deaths (Zhou et al., 2022), and its five-year survival rate is only 50%–60% (Troiano et al., 2018), with poor prognosis (Zhong et al., 2019). Currently, surgical treatment is typically used to directly remove the tumor, whereas adjuvant radiotherapy and chemotherapy are used to inhibit recurrence (Yang et al., 2020). Nonetheless, eliminating all cancer cells through surgery is challenging, resulting in a notable risk of local tumor recurrence (Kase-Kato et al., 2021). Additionally, radiotherapy and chemotherapy can cause severe side effects, placing considerable mental and financial stress on patients. Bone invasion has emerged as the most frequent complication associated with OSCC, primarily because of the unique anatomical interrelations between the oral soft tissue and jawbone (Chen et al., 2021). Therefore, for the treatment of OSCC, there is an urgent need to develop new therapeutic approaches to reduce side effects, inhibit postoperative tumor recurrence, and promote bone tissue regeneration at the bone defect site after surgery.

Recently, photodynamic therapy (PDT) has garnered considerable interest owing to its ability to selectively target tumors with minimal side effects and low costs (Liu et al., 2020; Yang et al., 2022). In PDT, photosensitizers are locally activated under irradiation to produce reactive oxygen species (ROS), inducing chemical damage that leads to tumor cell death (Benson et al., 2021). Photosensitizers used for photodynamic therapy include porphyrins, chlorine, bacterial chlorine, and phthalocyanine. Amphiphilic chlorin e6 (Ce6) is considered a promising photosensitizer for PDT. It is a degradation product of chlorophyll that is refined and modified using modern scientific and technological methods. Since its broad absorption (400 and 668 nm) matches well with the NaYF_4_ spectrum for the optimal generation of singlet oxygen, Ce6 offers the following advantages: rapid removal, high singlet oxygen production efficiency (Dou et al., 2015), near-infrared light excitation (Mikhail et al., 2010), and fluorescence imaging capability. To achieve accurate targeting of photosensitizers, reduce the amount of photosensitizer used, minimize side effects, avoid drug resistance (Shen et al., 2023), and enhance the therapeutic effect of PDT (Peng et al., 2020), the targeted delivery of Ce6 is highly desirable. The coupling of folic acid (FA) to target folate receptors expressed on tumor cells has emerged as a promising tool for targeted therapy. Folate receptors have been identified as key tumor biomarkers in numerous malignancies (Santra et al., 2005; Zhao et al., 2019). The cellular transport of folate is mediated by reduced folate carriers and/or folate-binding proteins. Studies have achieved successful drug delivery to OSCC cells via FA-mediated transport mechanisms (Fan et al., 2020). Both FA and Ce6 are connected to the surface of the nanoparticles via amide bonds.

However, the photosensitizers used in PDT often require a carrier for protection from degradation when transported to the intended treatment site. Lanthanide (Ln^3+^)-doped upconversion nanoparticles (UCNPs) have emerged as promising candidates for biological applications owing to their distinctive physicochemical characteristics (Liu et al., 2018; He et al., 2018). Through remote *in vitro* activation, NaYF_4_ effectively converts near-infrared light to the wavelength necessary to activate photosensitizers, thereby initiating PDT (De Groof et al., 2019; Kobayashi et al., 2022). Additionally, the ability of near-infrared light to penetrate normal tissues is significantly greater than that of ultraviolet or visible light, offer a greater potential for tumor treatment in PDT (Huang et al., 2020). However, owing to the coating of organic ligands produced during NaYF_4_ synthesis, it is insoluble in water, hindering its biological application (Yan et al., 2017). Researchers coated its surface with a layer of mesoporous silica (Mingzhou et al., 2022) to improve the loading rate of the photosensitizer (Zhang et al., 2016), resulting in good biosecurity. Therefore, NaYF_4_@SiO_2_ was selected as both a carrier and phototransducer with transmission and activation functions in PDT.

According to the current standard of care for OSCC, surgical resection, postoperative infection, or inadequate blood supply can lead to severe bone defects (Brennan et al., 2022). Therefore, it is necessary to repair bone defects after surgery. Over half of the reserves of magnesium ions, which are the second most prevalent intracellular cations in mammals (Lahav et al., 2021), are stored in the bones (Pouroutzidou et al., 2021). Magnesium-containing composite materials exhibit comparable biological activity to bone, and magnesium is degradable in the body, mainly releasing non-toxic magnesium oxide and Mg^2+^; during the degradation process, it can be completely excreted through the urine (Wong et al., 2019; Wang et al., 2020). More importantly, evidence shows that the supplementation of magnesium in the body can promote the growth of new bone (Armenise et al., 2020). Therefore, magnesium composites show great potential for application in bone defect repair.

To meet the dual clinical needs of bone defect repair and postoperative tumor recurrence prevention in OSCC, we developed a novel bifunctional bone replacement material with a sodium alginate (SA) hydrogel as the carrier matrix, doped with magnesium particles (MPs) and UCNPs. The uniform dispersion and stable release of the two functional particles were successfully achieved (Giri et al., 2012; Tan et al., 2023).

Preparation of NaYF_4_@SiO_2_/FA/Ce6 (UFC) and composite hydrogel (SUMg) and evaluation of osteogenic and tumor inhibition effects.

## 2 Materials and methods

### 2.1 Materials

UCNPs (supplied by Dalian Minzu University); concentrated hydrochloric acid and absolute ethanol (supplied by Jinzhou Medical University); and cetyltrimethylammonium bromide (CTAB), cyclohexane, NaOH, tetraethyl orthosilicate, acetic acid solution, 3-aminopropyltriethoxysilane, DMSO, CaCl_2_, and SA (Merck KGaA, Germany) were obtained.

Phosphate buffer solution (PBS), fetal bovine serum (FBS), hematoxylin and eosin (H&E), alizarin red S solution, an alkaline phosphatase (ALP) assay kit, a BCA assay kit, Calcin-AM/PI, and phalloidin-FITC were purchased from Solarbio (China). d-Minimum essential medium (d-MEM), α-minimum essential medium (α-MEM), and medium 1,640 were purchased from Hyclone (United States); penicillin–streptomycin solution from Thermo Fisher Scientific (United States); a cell counting kit-8 (CCK-8) from DOJINDO (China); and Triton X-100 from Sigma Aldrich (United States).

### 2.2 Synthesis of UFC

First, 8 mL of a 0.07 M CTAB aqueous solution was placed into a beaker, followed by the addition of 0.66 mL of UCNPs, and stirred for 2 h. Subsequently, cyclohexane was evaporated at 80 °C, after which 30 mL of water was incorporated, and 200 μL of a 0.1 M NaOH aqueous solution was added post-sonication for 15 min. Finally, 350 μL of tetraethyl orthosilicate and 1.2 mL of ethanol were introduced to the reaction mixture at a flow rate of 0.5 mL/h, allowing the system to mature at 35°C for 24 h. Following the reaction, ethanol was used to wash the NaYF_4_@mSiO_2_(CTAB) nanoparticles, which were then dried for 48 h. Then, 20 mg NaYF_4_@mSiO_2_(CTAB) nanoparticles were dispersed into 10 mL deionized water with 200 μL acetic acid and then added to 35 μL 3-aminopropyltriethoxysilane under agitation. The 3-aminopropyltriethoxysilane was hydrolyzed to form SiO_2_–(CH_2_)_3_-CH_2_, which was coated onto the surface of NaYF_4_@mSiO_2_. After high-speed centrifugation, the particles were dispersed into 40 mL MeOH, and then 400 μL concentrated hydrochloric acid was added to the system and reacted at 75°C for 48 h. After centrifugation, the precipitate was collected and washed twice with MeOH to yield NaYF_4_@mSiO_2_-NH_2_, effectively removing the CTAB molecules. Then, the particles were dispersed in DMSO, and the activated FA and Ce6 solutions were added, stirred overnight, centrifuged, and washed for collection.

### 2.3 Synthesis of SUMg

Next, 50 mg/mL calcium chloride solution and 5 mg/mL SA solution were dissolved in a water bath at 60°C and 80 Hz for 30 min. MPs and UFC were added to an SA solution and magnetically stirred for 30 min. SA solution was weighed out, and a calcium chloride solution was added to it to form a complex hydrogel. The samples were then freeze dried.

### 2.4 Basic characterization

The morphology and size of NaYF_4_@SiO_2_ and UFC particles were analyzed using transmission electron microscopy (Talos L120C G2, United States). The luminescence of the prepared UFC was measured using a 980 nm laser. The functional groups of UFC were characterized using Fourier transform infrared spectroscopy (Nicolet iS50, Thermo Fisher Scientific).

The swelling rate of the hydrogels was determined by measuring the changes in their wet weight. First, the SUMg was completely lyophilized and soaked in a PBS solution (1 mL) at 37°C. The hydrogel was then removed from the solution at specific intervals, and excess water was removed using a filter paper. Subsequently, the weight of the SUMg sample was recorded to calculate the swelling rate using the following formula: swelling ratio = Wt/W0, where Wt is the weight of the hydrogel after incubation with the PBS solution, and W0 is the original weight. The rheological properties of SUMg were analyzed using a DHR-2 machine (TA Instruments, United States). The samples were prepared in cylindrical shapes, each with a thickness and diameter of 8 mm, and compression tests were carried out at a rate of 10 mm/min. The injectability, malleability, and self-healing properties of SUMg were verified using a general-purpose experimental machine and a special heart-shaped mold. First, a syringe was used to draw 1 mL of the SUMg sample for injection, and then, a universal experimental machine was set up to add SUMg to the heart-shaped mold at a speed of 50 mm/min. The SUMg was cut and observed. To investigate the degradability of SUMg, each group of samples was weighed (W0), immersed in a test tube containing 10 mL PBS, and placed on a constant-temperature shaking table at 37°C. Samples were collected at predetermined time points and washed three times with deionized water. The samples were then dried in a fume hood and weighed (wt%). The degradation rate was measured as follows: degradation rate (%) = (W0–Wt)/W0 ×100%. The hydrogel morphologies were analyzed using field-emission scanning electron microscopy (Zeiss Gemini 300, Germany). SA and SUMg were analyzed by FTIR spectroscopy. To study the morphological characteristics of the samples, an SU8010 scanning electron microscope (Hitachi, Japan) was used, and the acceleration voltage was set to 5 kV. Elemental analysis was also performed.

### 2.5 Singlet oxygen detection

By utilizing 9,10-anthracenediyl-bis(methylene) dimalonic acid (ABDA) as a molecular indicator to assess the production of singlet oxygen (1O_2_), we indirectly evaluated the therapeutic effectiveness of PDT induced by UFC when excited by near-infrared light. The UFC was positioned at the bottom of a quartz colorimeter, into which 2 mL of a 0.2 mg/mL ABDA solution was gradually introduced along the bottom. The same time intervals (10, 20, 30, 40, 50, and 60 min) were selected for UV–visible light measurement using 980 nm laser irradiation.

### 2.6 Biocompatibility evaluation of UFC

The human oral squamous cell line HSC-4 was maintained in d-MEM supplemented with 10% FBS and 1% streptomycin and penicillin. Mouse osteoblastic precursor cells (MC3T3-E1) were seeded into 96-well plates and incubated for 24 h. UFC was immersed in complete d-MEM at the following concentrations: (1) 0 μg/mL, (2) 100 μg/mL, (3) 200 μg/mL, (4) 400 μg/mL, (5) 800 μg/mL, and (6) 1,000 μg/mL. HSC-4 cells were treated with the prepared extract instead of complete medium. After 24 and 48 h, 10 μL CCK-8 reagent and 90 μL medium were added, respectively, and samples were cultured at 37°C without light for 2 h. Optical density was measured at 450 nm using a microplate reader.

### 2.7 Evaluation of UFC targeting effect *in vitro*

HSC-4 and HOK cells were used as models to detect the targeting efficiency of the preprogrammed nanoparticles. Cells were inoculated in 24-well plates and cultured to 80% confluence. Cells were then co-cultured separately with UFC at 1,000 μg/mL. After incubation for 24 h, cells were washed three times with PBS, fixed with 4% formaldehyde, and observed under an inverted fluorescence microscope with an external 980 nm light source.

### 2.8 Role of UFC in PDT *in vitro*

HSC-4 cells were prepared in a single-cell suspension, placed in 96-well plates, and incubated at 37°C with 5% carbon dioxide for another 24 h. The experimental design included the following groups: control, NIR, NaYF_4_, NaYF_4_+NIR, UFC, and UFC + NIR + NIR with three replicates per group. The cells were treated in turn according to the groups, and after co-incubation for 24 h, the nanoparticles not taken up by the cells were washed off, and fresh medium was added. The laser-irradiated group received a 980 nm near-infrared laser treatment at a power density of 1 W/cm^2^ for 5 min, after which the cells were incubated for an additional 24 h. After adding 50 μL CCK-8 reagent, the cells were incubated away from light for 2 h. The optical density was determined using an enzyme marker at a 450 nm wavelength.

### 2.9 Biocompatibility evaluation of SUMg

MC3T3-E1 cells were maintained in α-MEM supplemented with 10% FBS and 1% streptomycin and penicillin. Cells were then seeded in 96-well plates and incubated for 24 h (1) SA, (2) SA/NaYF_4_, (3) SA/NaYF_4_+NIR, (4) SA/NaYF_4_/5%Mg, (5) SA/NaYF_4_/10%Mg, and (6) SA/NaYF_4_/15%Mg were immersed in complete α-MEM and incubated at 37 °C for 24 h. Instead of the complete medium, MC3T3-E1 cells were treated with the prepared extract. After 1, 2, and 3 days, 10 μL CCK-8 reagent and 90 μL of medium were introduced, followed by a 2 h incubation at 37°C in the absence of light. Optical density was measured at 450 nm using a microplate reader.

MC3T3-E1 cells in good condition were collected and spread on 24-well plates. After incubation for 24 h, extracts were added according to the groups and cultured for 24 h. A working solution comprising Calcin-AM/PI double dye was then introduced, and cells were incubated in the dark for 15 min. Changes in cell color were examined using an inverted fluorescence microscope.

MC3T3-E1 cells were inoculated into 24-well plates and incubated for 24 h, and then the following extracts were added: (1) control, (2) SA/UFC/5%Mg, (3) SA/UFC/10%Mg, and (4) SA/UFC/15%Mg. The cells were cultured for 24 h and fixed in 4% paraformaldehyde for 30 min. Then, cells were stained with 5 μg/mL of phalloidin for 60 min. Next, cells were stained with 200 μL of DAPI for 10 min. Cytoskeleton morphology was observed under a microscope after washing with PBS.

### 2.10 Osteogenic differentiation evaluation of MC3T3-E1

MC3T3-E1 cells were plated in 6-well plates and incubated for 24 h. Subsequently, the osteogenic induction medium was prepared by adding 10% FBS, 100 nM dexamethasone, 50 μM ascorbic acid, and 10 mM glycerol β-phosphate. After culturing for 7 and 14 days, the culture solution was carefully removed, and the pore plate was gently cleaned twice with PBS. The cells were then treated with an ALP quantitative kit and a BCIP/NBT ALP chromogenic kit according to the manufacturer’s instructions, and ALP activity was determined and quantified. The osteogenic differentiation potential of the MC3T3-E1 cells was evaluated in each treatment group. To further assess osteogenic differentiation, an alizarin red assay was conducted to evaluate extracellular matrix mineralization. MC3T3-E1 cells were cultured using the same procedure for 21 days, followed by three washes with PBS. The cells were then fixed at room temperature with 4% paraformaldehyde for 30 min and subsequently stained with alizarin red S solution. A volume of 3 mL of 0.1% alizarin red dye solution was added to each well and incubated at 37°C for 30 min. After washing with PBS, the mineralized nodules were examined under an inverted fluorescence microscope and photographed.

### 2.11 Evaluation of UFC anti-tumor effect *in vivo*

Four-week-old female BALB/c nude mice were acquired from Liaoning Changsheng Biotechnology Co., Ltd. All animal handling procedures for *in vivo* treatment adhered to the protocol established by the Experimental Animal Center of Jinzhou Medical University and were approved by the Animal Ethics Committee of the same institution. After a week of observation in female BALB/c mice, 0.1 mL HSC-4 (2 × 10^6^ cells/mL) were injected subcutaneously into the right forelimbs of nude mice to create a tumor model for subsequent investigations. When the tumor size reached 100 mm^3^, the tumor-bearing mice were randomly assigned to the (1) laser, (2) UFC injection, or (3) UFC injection + 980 nm laser group. Following injection, the tumor was exposed to a 980 nm laser (4 W cm^−2^, 5 min). The tumor size and body weight of the nude mice were recorded daily. The tumor volume (V) was determined using the formula V = L × W^2^/2, where L and W represent the tumor length and width, respectively. Following the experiment, organ (heart, liver, spleen, lung, and kidney) and tumor tissue samples were collected. The collected specimens were fixed in 4% paraformaldehyde and subsequently embedded, dried, and stained with H&E for microscopic analysis.

### 2.12 *In vivo* osteogenic effect of SUMg

To verify the overall osteogenic effect of the material, each group of hydrogels was prepared before surgery; particle size and shape were controlled to meet the intraoperative requirements, and the hydrogels were subjected to strict ultraviolet disinfection. Four-week-old female Sprague–Dawley rats were acquired from the Experimental Animal Center of Jinzhou Medical University. All *in vivo* animal procedures were conducted according to the authorized protocols set by the Experimental Animal Center of Jinzhou Medical University. The study protocol was reviewed and approved by the Animal Ethics Committee of the Jinzhou Medical University. After a week of observation, the rats were anesthetized via pentobarbital injection. The fur covering the skull was removed and the skin disinfected, and then a cut was made along the central axis of the skull to remove the fascia and reveal the skull. Under normal saline cooling, circular hollow high-speed drilling was used to create a round full-layer defect with a diameter of 5 mm on one side of the skull. The rats with skull defects were randomly divided into three groups: (1) SA, (2) SA+UFC, and (3) SA+UFC+10%Mg. After implantation, the incisions were closed with sterile sutures. The rats were euthanized at 4 W after surgery for sampling; the organs (heart, liver, spleen, lung, and kidney) were collected for biosafety evaluation, and skull specimens were collected. Post-fixation skull bone regeneration was assessed using micro-CT (Aloka lattheta CTT-200, Hitachi), with 2D X-ray images processed into 3D visuals utilizing the 3D image processing software CTvox 3.0 (Bruker, Germany). The bone trabeculae and bone volume fractions (BV/TV) were analyzed. After dehydrating and embedding the skull samples, staining was performed using H&E and Masson’s trichrome, followed by microscopic examination.

### 2.13 Statistical analysis

One-way analysis of variance (ANOVA) was performed using SPSS Statistics 25 software (IBM, Armonk, NY, United States). Results are presented as mean ± standard deviation (SD), with a significance threshold of P < 0.05 considered statistically significant (****P < 0.0001, ***P < 0.001, **P < 0.01, *P < 0.05).

## 3 Results and discussion

### 3.1 Characterization of UFC

**FIGURE 1:**
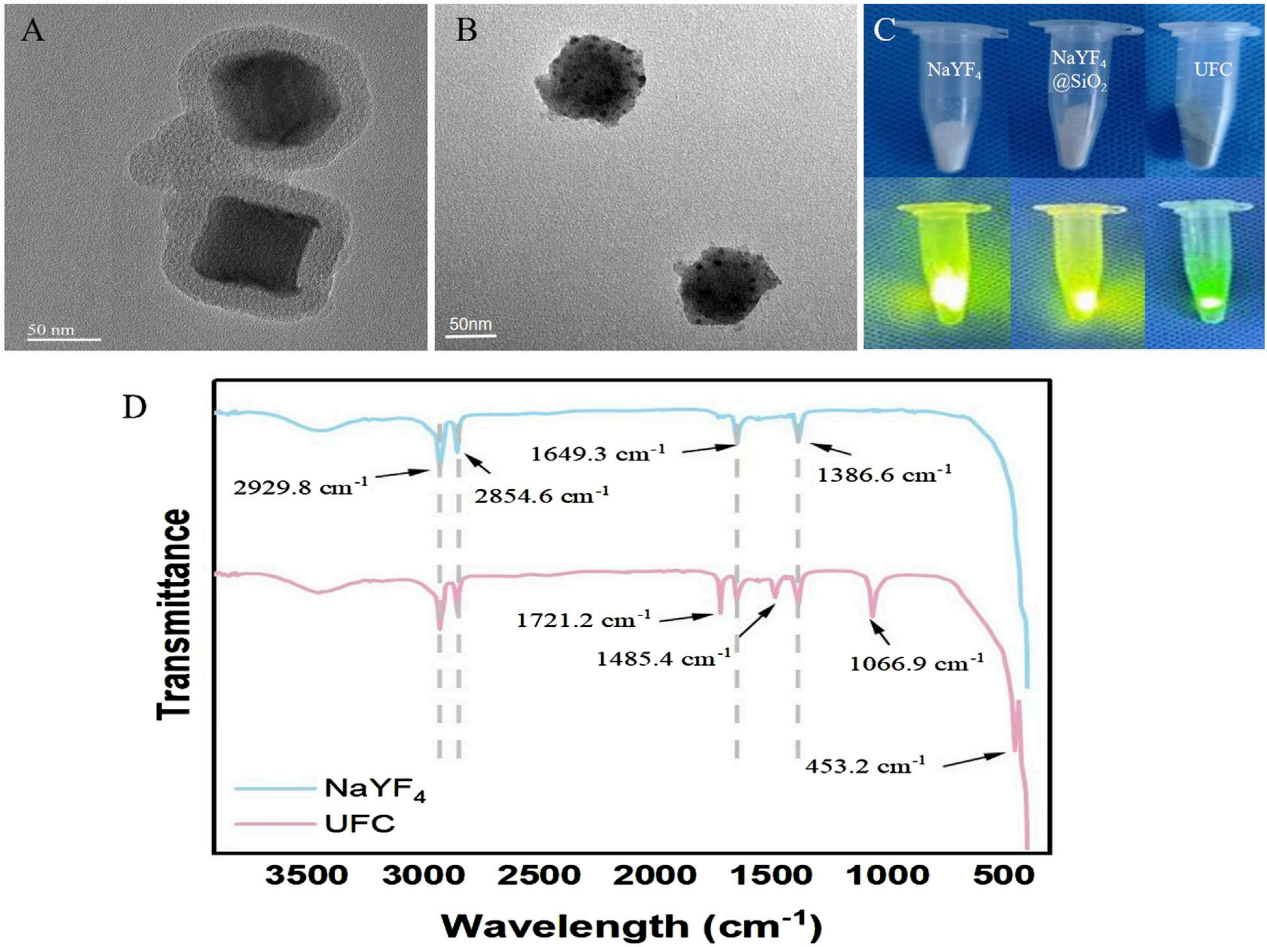
Characterization of UFC. **(A)** TEM image of NaYF_4_@SiO_2_. **(B)** TEM image of NaYF_4_@SiO_2_/FA/Ce6(UFC). **(C)** NaYF_4_, NaYF_4_@SiO_2_(UFC) macro image and 980 nm laser excitation luminescence image. **(D)** Fourier spectral images of NaYF_4_ and UFC.

Figure 1A shows that after NaYF_4_ was coated with SiO_2_, the particle size was approximately 80 nm, and the particles were randomly dispersed into a single layer, in which NaYF_4_ was still clearly visible in its hexagonal state. Figure 1B shows the successful encapsulation of Ce6 and FA in the NaYF_4_@SiO_2_ silicon shell. Figure 1C shows that when NaYF_4_ was coated with SiO_2_, FA, and Ce6, the surface of the macro-nanoparticles showed clear color changes from pure white to dark green. In addition, under the excitation of a 980 nm laser, the luminous intensity of each group of powders was visibly weakened, and the luminescence of NaYF_4_ was hindered, indicating successful packaging and surface modification. The Fourier spectra shown in Figure 1D were used to analyze the functional groups of the nanoparticles. The NaYF_4_ spectra confirmed that 2,929 cm^–1^ and 2,853 cm^–1^ corresponded to the stretching vibration absorption peaks of the long alkyl methylate chain of the UCNPs, and 1,650 cm^–1^ and 1,387 cm^–1^ corresponded to the stretching vibration absorption peaks of the C=C double bond. In the UFC spectrum, the newly emerged band at 1,067 cm^–1^ belonged to the absorption vibration of Si–O, indicating successful silicon encapsulation, and the newly emerged band at 1,485 cm^–1^ belonged to the tensile vibration of -NH_2_, indicating that the amino group was successfully connected to the silicon shell on the surface of the UCNP. Meanwhile, the new Ce6 wave peak can be seen at 452 cm^–1^, and the absorption vibration peak of C=O at 1720 cm^–1^ indicates that the FA connection was successful.

### 3.2 Characterization and physical properties of SUMg

**FIGURE 2:**
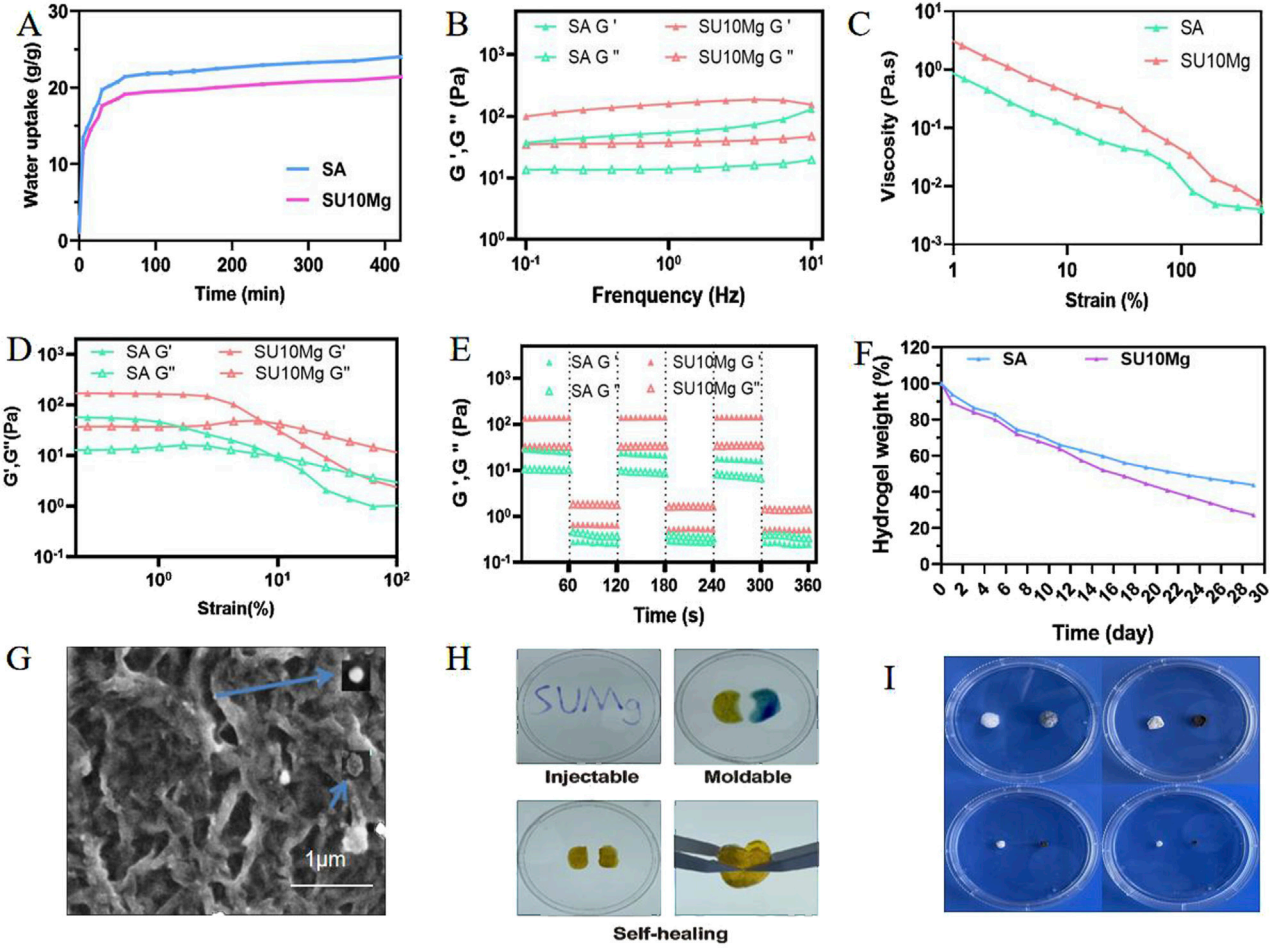
Characterization and physical properties of SUMg. **(A)** Swelling test results of SA and SUMg. **(B)** Dynamic frequency scan results of SA and SUMg. SA G′ and SUMg G′ are solid gel states and represent the energy storage moduli. SA G″ and SUMg G″ are gel liquid states and represent the loss moduli. **(C)** Shear thinning test results. **(D)** Strain scanning experiment results of SA and SUMg. SA G′ and SUMg G′ are solid gel states and represent the energy storage moduli. SA G″ and SUMg G″ are gel liquid states and represent the loss moduli. **(E)** Dynamic amplitude measurement results of SA and SUMg. SA G′ and SUMg G′ are solid gel states and represent the energy storage moduli. SA G″ and SUMg G″ are gel liquid states and represent the loss moduli. **(F)** Degradation weight rate of the hydrogel. **(G)** SEM images of SU10 Mg. **(H)** Injectable, moldable, and self-healing assay results for SUMg. **(I)** SUMg 1–4 weeks degradation graph.

**FIGURE 3:**
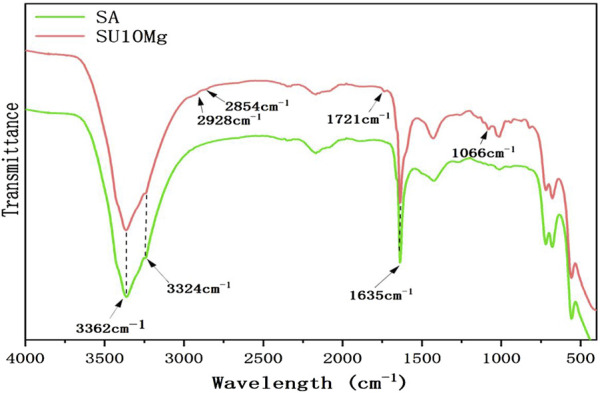
Chemical characterization of SUMg.

**FIGURE 4:**
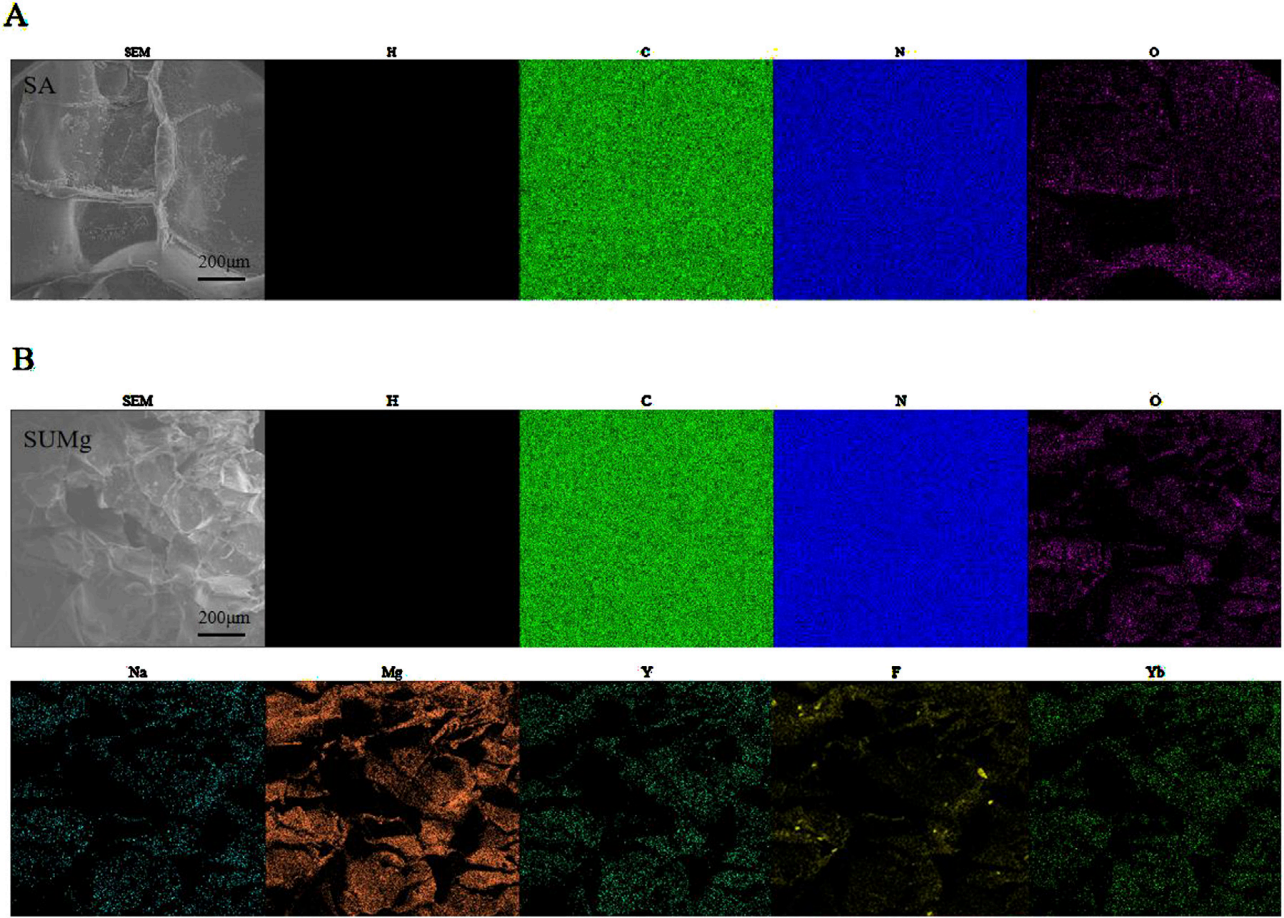
Physical characterization of SUMg. **(A)** Element mapping of SA (H, C, O, N). **(B)** Element mapping of SUMg (H, C, O, N, Mg, Na, Y, F, Yb).

As shown in Figure 2A, the swelling rates of both SA and SUMg increased rapidly within 5 min, indicating that the lyophilized hydrogel rapidly absorbed PBS during this period, gradually expanded, and reached swelling equilibrium at 7 h. The prepared hydrogels exhibited good water absorption, and the swelling peak value of the SUMg group was lower, which may be due to the addition of UFC and MPs. However, the successful addition of both did not affect the swelling performance. Figure 2C shows that under external force, both groups of gels underwent shear deformation, and their values decreased, suggesting successful degradation. As shown in Figure 2D, the intersection points of energy storage and loss moduli exist in the curves of SA G′ and SA G″ and of SU10 Mg G′ and SU10 Mg G″. These gel crosslinking points suggest good glue-forming properties. In addition, the mechanical strength of the SU10 Mg group was better owing to the addition of MPs, as confirmed in Figure 2B. As shown in Figure 2E, both gels broke after 60 cycles of external stimulation but recovered in the next cycle, indicating good self-healing properties. Moreover, during the first 10 days, the degradation rates of the hydrogels in the two groups were roughly the same (Figures 2F, I). However, after 10 days, owing to the exposure to MPs and UFC in SUMg, the pores in the hydrogels increased, which may also explain why the cells were fully extended. Moreover, the bone formation effect of SUMg was better in subsequent experiments. As shown in Figure 2H, SUMg exhibited good fluidity and gelatinization, being gelatinized by injection and transformed into a plastic with ideal properties. With an external shear force, the SUMg still healed after a few minutes and did not break under a slight external force. The SUMg scanning electron microscope image in Figure 2G shows the successful loading of Mg particles and UFC into the SA hydrogel. The Fourier spectrum was used to analyze SA and SUMg. As shown in Figure 3, the characteristic peak of -OH appeared at 3,362–3,244 cm^–1^ for SA, and the characteristic peak of C–O in the tensile vibration appeared at 1,635 cm^–1^. In SUMg, new UFC characteristic peaks were observed, such as the tensile vibration characteristic peaks at 2,928, 2,854, 1,721, and 1,066 cm^–1^, indicating that UFC was successfully added to SUMg. As for the added MPs, because they does not undergo structural changes in SA, their spectrum does not change (Adhikari et al., 2019). Further elemental analysis confirmed that the MPs were successfully added. Figure 4 shows that Mg, Na, F, Y, etc., are present in the UFC.

### 3.3 Biocompatibility of UFC

**FIGURE 5:**
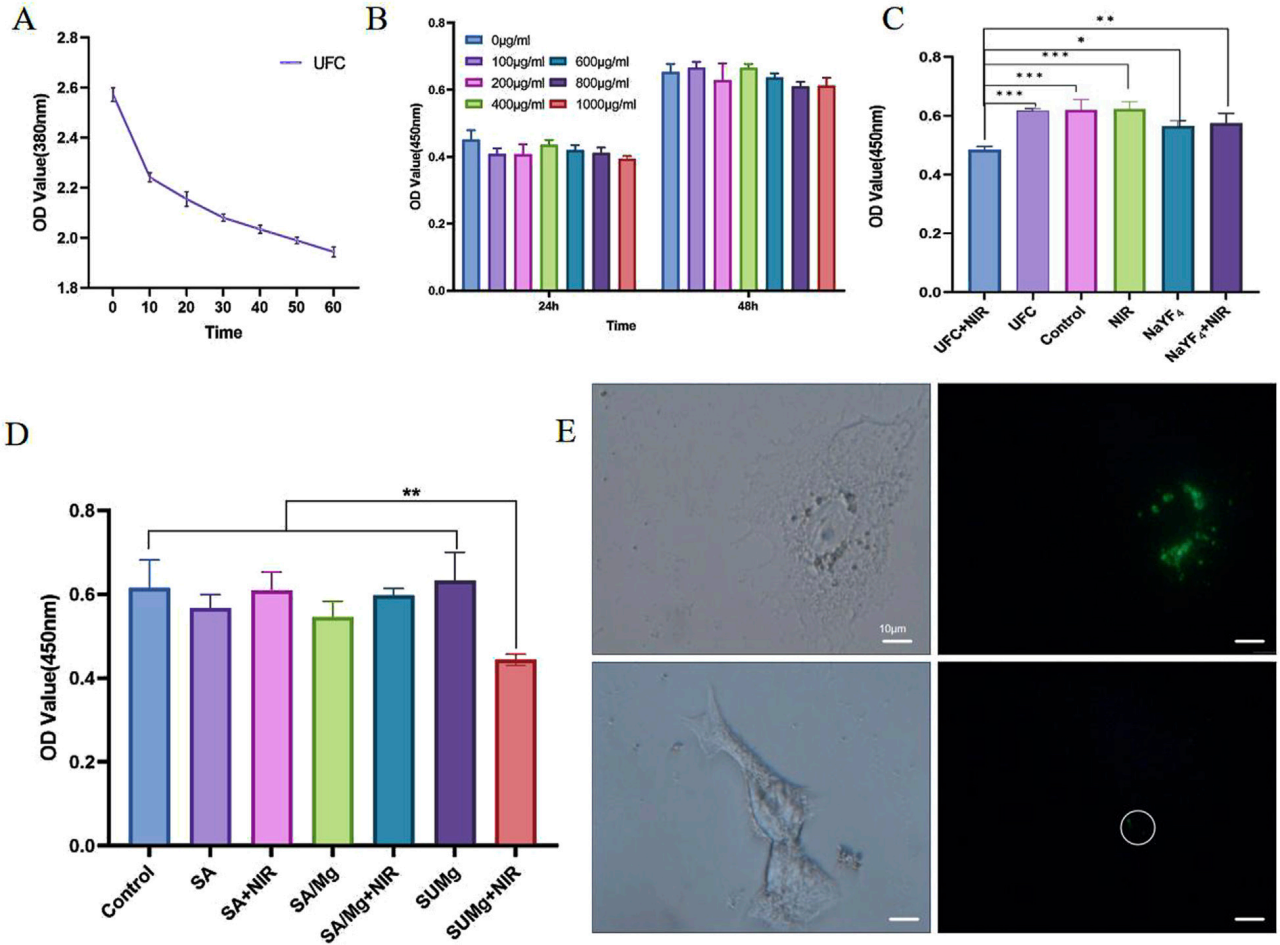
*In vitro* evaluation of UFC. **(A)** 1O_2_ release curve of UFC under 980 nm excitation. **(B)** Biotoxicity of UFC **(C)** Photodynamic tumor cell inhibition effect of UFC **(D)** Photodynamic tumor cell inhibition effect of SUMg **(E)** Squamous cell carcinoma and oral keratinocyte uptake effects of UFC. Data are represented as means ± standard deviation (SD) for three biological replicates (n = 3). *p < 0.05, **p < 0.01, ***p < 0.001.

The most basic requirement for the application of nanoparticles in biomedicine is good biocompatibility. Biocompatibility refers to the ability of a biomaterial to perform its desired function in the implanted tissue without causing any unwanted local or systemic response in the host while inducing the most appropriate beneficial cellular or tissue response (Hassanzadeh et al., 2021). We wanted the developed material to be non-toxic and to inhibit cancer cells under near-infrared irradiation. To verify this, we used the CCK-8 assay to determine the toxicity of UFC. Because of the high cytotoxicity of NaYF_4_ nanoparticles, which are encapsulated with organic ligands during the synthesis process (Sarkar et al., 2016), making them insoluble in water and compromising their biological applications, a mesoporous silica layer is typically applied to their surfaces. This coating not only enhances the loading efficiency of the photosensitizer but also ensures good biocompatibility. d-MEM was used to establish UFC media with concentrations of 0, 100, 200, 400, 600, 800, and 1,000 μg/mL. HSC-4 cells were cultured after treatment for 24 and 48 h. HSC-4 activity remained high at all the detected concentrations after 24 h. After 48 h, the tolerance of HSC-4 cells changed with the increase in UFC concentration, but at the maximum concentration of 1,000 μg/mL, the survival rate of the cells was still very high and not statistically different from that of the control group, confirming non-toxicity (Figure 5B).

Numerous studies have shown that UFC doped in SUMg is released by the degradation of the material. The released UCNPs are non-toxic, metabolized by the liver and spleen, excreted in urine, and do not break down in the body (Cao et al., 2013). However, the composite hydrogel platform SUMg was doped with SA and MPs, of which the former, a common tumor drug-carrying platform (Yang et al., 2024), had almost no effect on tumors. The content of magnesium ions released by MP degradation was 5%–15% of SUMg, reaching a sufficient concentration to promote osteoblast formation (Wang et al., 2014) but not to affect tumor cells (Zhou et al., 2021). Therefore, the influence of these two factors on the material can be excluded. Further supporting this conclusion, Figure 5D shows that the influences of NIR, SA, and MPs can all be excluded.

Further *in vitro* experimental cytotoxicity studies confirmed that under 980 nm laser excitation, the addition of UFC successfully destroyed tumor cells. According to the CCK-8 assay results (Figure 5C), the cells in each group showed different growth trends 48 h after treatment. The survival of HSC-4 cells co-cultured with UFC nanoparticles and exposed to light alone was unaffected, indicating that the nanoparticles and upconverted UV light did not cause cell death. In the UFC group stimulated by the 980 nm laser, cell survival was greatly reduced. Thus, the PDT effect was successfully induced. The main principle of anti-tumor PDT is that 1O_2_ oxidation destroys organelles in the cell, causing tumor cell apoptosis. However, nanoparticles with photodynamic anti-tumor functions need to be effectively taken up by tumor cells to ensure the release of 1O_2_, destruction of organelles, and anti-tumor effect.

### 3.4 ROS release detection by UFC

As 1O_2_ could not be detected directly, we used ABDA as the probe molecule to detect 1O_2_ production indirectly. The principle is that ABDA exhibits UV–visible absorption between 300 and 425 nm, with maximum absorption at 380 nm. In the presence of 1O_2_, ABDA generates internal peroxides, causing its absorption peak to decline at 380 nm. Therefore, the level of 1O_2_ is proportional to the decrease in the intensity of the UV–vis absorption peak of ABDA. The results shown in Figure 5A confirm this principle. With the extension of the 980 nm laser irradiation time, the peak UV absorption at 380 nm gradually decreased, and the decline rate was highest within 10 min, possibly because Ce6 loaded in the UFC was fully activated within 10 min and ROS produced remarkable effects.

### 3.5 Effect of UFC uptake

As UCNPs have good photostability and low autofluorescence and are deeply penetrated by near-infrared excitation, they have good application prospects for *in vitro* and *in vivo* PDT. In addition to the important therapeutic role of UCNPs, their upconversion imaging function can be used to monitor the location and enrichment of nanoparticles in tumor tissues. Therefore, to further investigate the role of UFC in tumor cells, we incubated UFC with HSC-4 and HOK (oral keratinocytes) cells for 24 h and then observed cell uptake under a standard microscope with an external 980 nm laser. As shown in Figure 5E, we found that the uptake effects of HSC-4 and HOK on UFC differed greatly. This may be due to the tumor-specific labeling of folate receptors on the surface of HSC-4, which specifically recognizes the FA on the UFC surface, whereas HOK cells only engulf UFC through simple endocytosis.

### 3.6 Biocompatibility of SUMg

**FIGURE 6:**
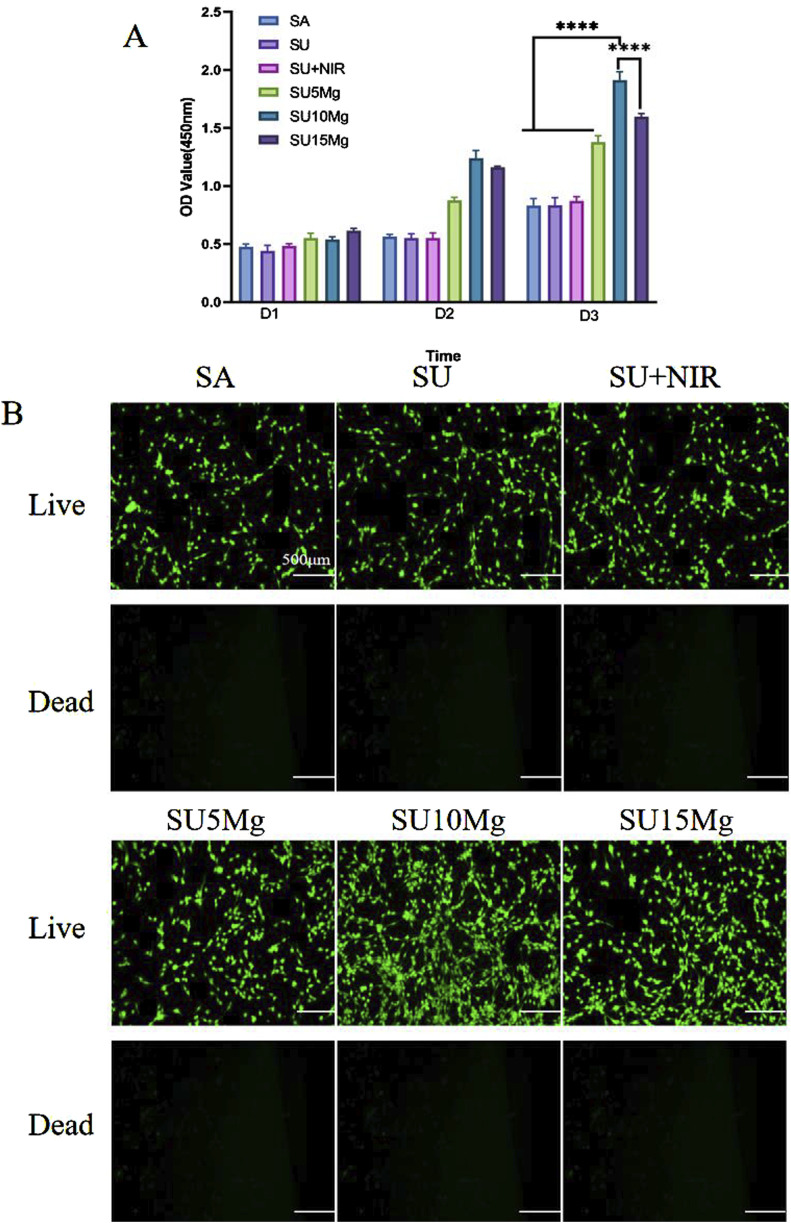
SUMg biosafety evaluation. **(A)** Biosafety of SUMg. **(B)** Fluorescent live/dead staining of osteogenic progenitor cells treated with SUMg. Data are represented as means ± SD for three biological replicates (n = 3). ****p < 0.0001.

Numerous studies have indicated that incorporation of Mg ions enhance cell adhesion and promotes cell proliferation. Our findings (Figure 6A) from the CCK-8 assay indicated that the cells in all groups displayed strong growth on the first day. However, by the second day, the proliferation observed in the SA, SU, and SU+NIR groups was less pronounced than that in the group with added magnesium particles. On day 3, this trend was more pronounced, particularly in the SU10 Mg group. When the concentration of magnesium particles was set at 10%, a notable enhancement in cell proliferation was observed, outperforming the two other concentrations of magnesium complex hydrogels. The hydrogel with a 10% magnesium particle concentration exhibited a suitable magnesium ion concentration and release rate. The 5% Mg concentration may not achieve the optimal release level of Mg^2+^, whereas a 15% concentration may lead to an excess of Mg ions, potentially causing a shift in the medium’s pH that would hinder cell growth and adhesion. Notably, the biocompatibility of the hydrogel remained good even after the addition of NaYF_4_ or the introduction of UV light for a short time.

We further verified the biocompatibility by live/dead staining, and as shown in Figure 6B, none of the materials in any group caused MC3T3-E1 cell death. The growth trend of the cells in each group matched the CCK-8 results.

**FIGURE 7:**
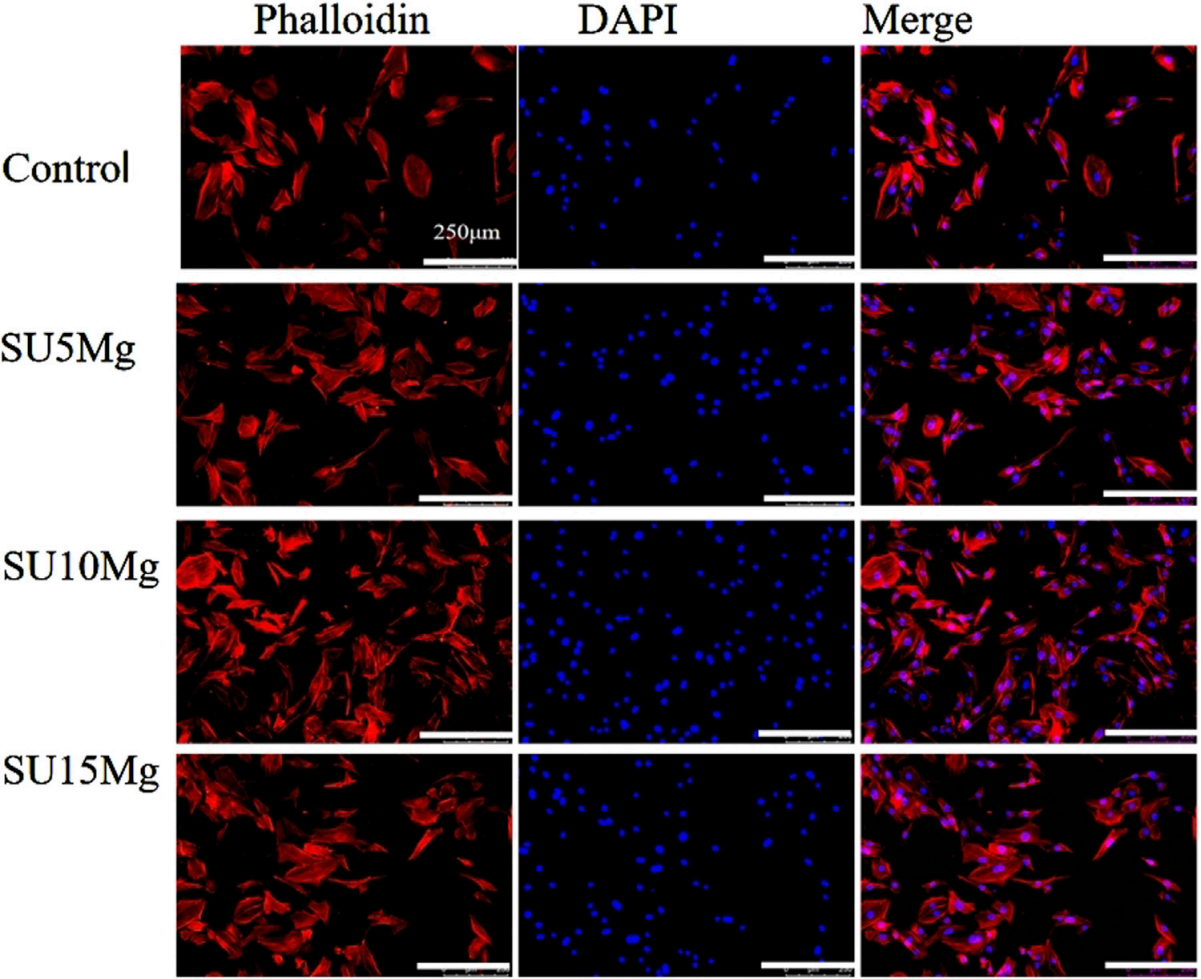
Promoting effect of SUMg on cell extension. Cytoskeleton fluorescence images of MC3T3-E1 cells stained with DAPI–phalloidin. There were three biological replicates (n = 3).

To further investigate the effect of SUMg on MC3T3-E1 differentiation, we observed the number and morphology of the cells in each group under a microscope (Figure 7). The remaining three groups showed higher cell counts than the control group. Moreover, the SU10 Mg group had the best cell morphology, largest cell spreading area, best cell growth conditions, most complete cell particles, and best connections with neighboring cells, indicating its superior effect on the extension of MC3T3-E1 cells.

### 3.7 Impact of SUMg on the osteogenic differentiation of MC3T3-E1 cells

**FIGURE 8:**
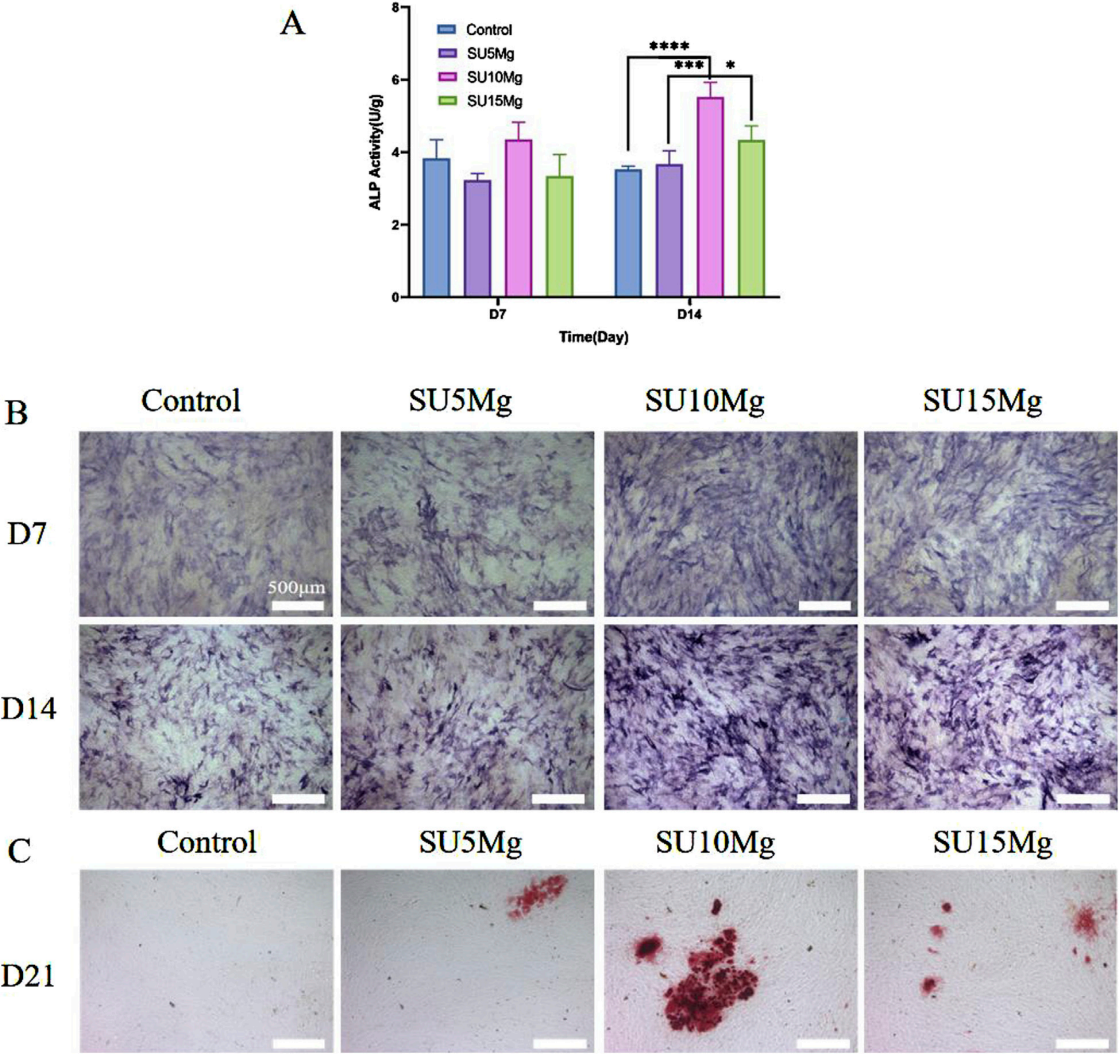
Impact of SUMg on the osteogenic differentiation of MC3T3-E1 cells. **(A)** Semi-quantitative results and ALP activity in of MC3T3-E1 cells on days 7 and 14 of hydrogel culture in each group. **(B)** ALP staining results of hydrogels of each group incubated with MC3T3-E1 cells on day 7 and day 14. **(C)** Alizarin red S staining of mineralized nodules on day 21. Data are represented as means ± SD for three biological replicates (n = 3). *p < 0.05, ***p < 0.001,****p < 0.0001.

ALP is widely acknowledged as a preliminary indicator of osteogenesis. The experiments mentioned earlier showed that the SU10 Mg hydrogels had better biocompatibility than the other groups; thus, the experiments on osteogenic differentiation focused on this specific material. In addition, because there was no significant difference between the SA, SU, and SU+NIR groups, only the SU group was selected as a representative in subsequent experiments. A semi-quantitative assessment of ALP levels indicated that the SU10 Mg group exhibited the highest level of enzyme activity (Figure 8A). Moreover, on days 7 and 14, the SU10 Mg group displayed more intense ALP staining than the other experimental groups, corroborating the semi-quantitative ALP data (Figure 8B). After 21 days of culture with MC3T3-E1 cells, the positive alizarin red staining area in the SU10 Mg group was significantly larger than that in the control group (Figure 8C). This implies that Mg^2+^ promotes the development of calcified nodules during the later stages of bone formation and further suggests that the mineralization of the matrix in the SU10 Mg group exceeds that in the groups with 5% and 15% MPs. Consequently, a suitable concentration of magnesium ions appears to promote the osteogenic differentiation of MC3T3-E1 cells. Overall, these results highlight the ability of SU10 Mg to enhance both cell proliferation and differentiation.

### 3.8 *In vivo* evaluation of tumor inhibition

**FIGURE 9:**
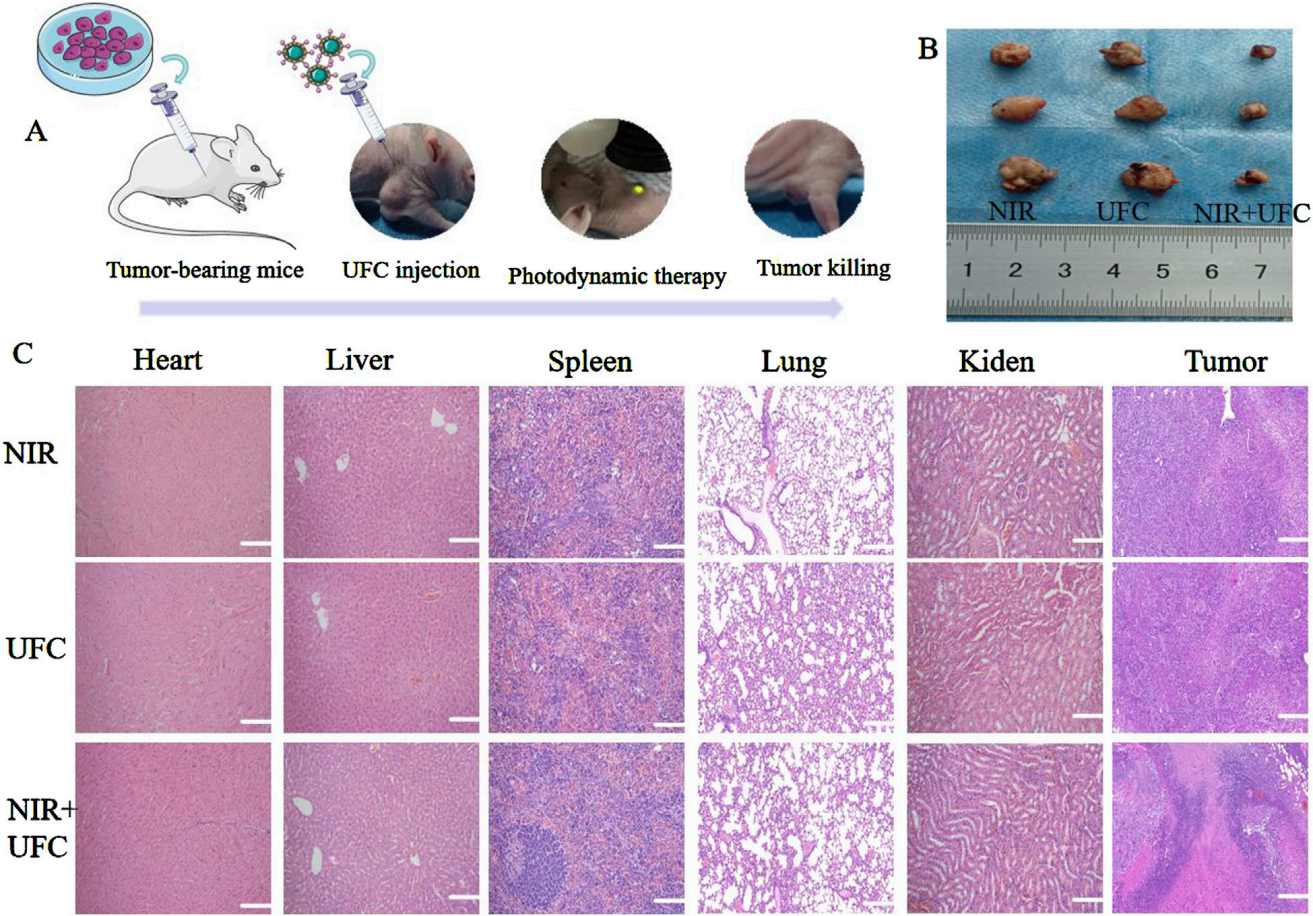
*In vivo* evaluation of tumor inhibition. **(A)** Experimental process diagram. **(B)** Typical image of a Balb/c nude mouse during treatment. **(C)** Histological evaluation of vital organs. There were three biological replicates (n = 3).

Tumor recurrence is a common complication of tumor treatment, resulting in a considerable psychological burden and economic pressure on patients. The timely suppression of tumor recurrence after surgery and promotion of bone defect repair are urgent challenges to consider when developing OSCC implant materials. Notably, because tumor recurrence does not stem from bone defects, the hydrogels and MPs were excluded from the *in vivo* tumor studies. The *in vitro* tumor treatment results indicated that the tumor probe exhibited significant inhibitory effects. Building on this, we investigated the effect of the tumor probe on OSCC *in vivo*. The tumor size and weight were measured daily as part of the experimental protocol. Neither the laser nor the UFC group showed significant inhibition of tumor growth. In contrast, the UFC+NIR group demonstrated a substantial inhibition of tumor growth by the 14th day, which was validated by *in vitro* investigations, suggesting the PDT capabilities of UFC. No significant weight loss or abnormal behavior was observed in any group during the experiment. We then performed tissue analysis of the tumor and other major organs for further studies. After treatment, H&E staining revealed no cell damage in the tumor, heart, liver, spleen, lung, or kidney across all groups. Notably, no changes were observed in the tumors, and the organs appeared largely unaffected by the treatment (Figure 9).

### 3.9 Bone repair *in vivo*

**FIGURE 10:**
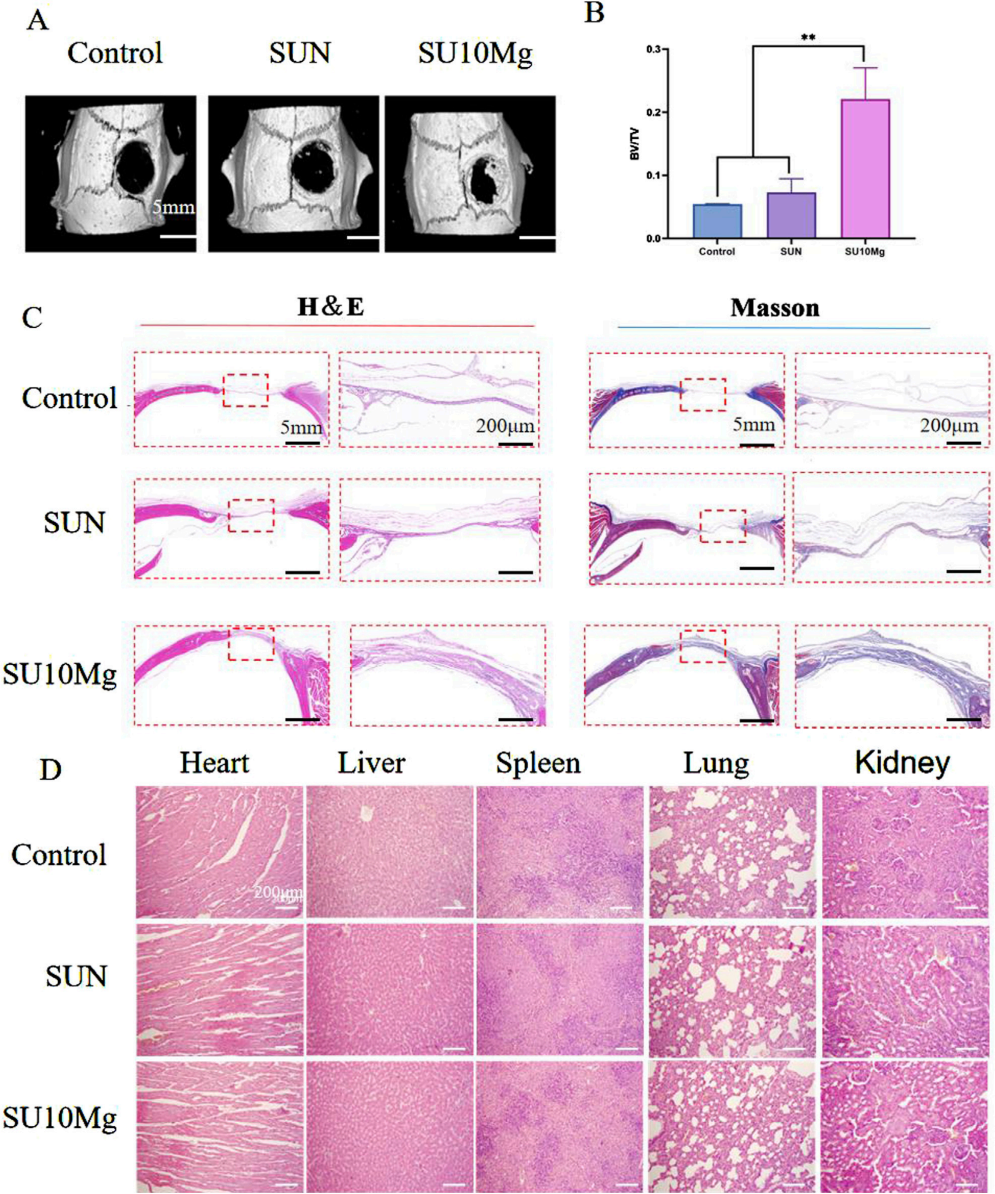
*In vivo* evaluation of osteogenic promotion. **(A)** Four-week postoperative micro-CT 3D reconstruction of the skull. **(B)** Postoperative skull bone tissue volume/total tissue volume (BV/TV). **(C)** H&E and Masson’s trichrome staining for the evaluation of bone regeneration. **(D)** H&E staining to observe morphological changes in heart, liver, spleen, lung, and kidney tissues. Data are represented as means ± SD for three biological replicates (n = 3). **p < 0.01.

Postoperative bone defects are frequent surgical complications for OSCC. Consequently, with the aim of combined bone defect repair and tumor suppression, we investigated the potential of composite hydrogels containing MPs to facilitate the repair of postoperative bone defects. Based on the *in vitro* experiments, the promoting effect of the composite hydrogel on bone defect regeneration *in vivo* was further verified. Because a 10% MP concentration was confirmed *in vitro* as the most suitable concentration for promoting bone repair, *in vivo*, only SU10 Mg treatment was selected for the experimental group, while the influence of SA, UFC, and near-infrared light was excluded, and the SU+NIR group was selected as the control group. Different compositions of the composite hydrogel were implanted into the skull defect, and after 4 weeks of culture, the skull was extracted for evaluation using micro-CT (Figure 10A). By the fourth week after surgery, bone formation was observed in all defect areas; however, the SU10 Mg group demonstrated considerable new bone formation surrounding the defect, with the highest fusion rate. Conversely, the results for the first and second groups were less favorable, echoing previous findings from cell experiments. The results were also confirmed by H&E and Masson’s trichrome staining of the skulls (Figure 10C). The quantitative analysis presented in Figure 10B shows that the BV/FV value for the SU10 Mg group was notably higher than those of the other two groups, highlighting its strong potential for bone integration and the promotion of bone defect healing. Additionally, H&E staining of the heart, liver, spleen, lungs, and kidneys of the experimental rats demonstrated no cell damage across the groups (Figure 10D). The results indicate that the treatment had minimal effects on the organs in all groups.

## 4 Conclusion

In this study, we successfully created a drug delivery system with a feasible design. The local release and delivery of magnesium ions were mediated by the degradation of the SA hydrogel. MC3T3-E1 cells cultured *in vitro* with SU10 Mg exhibited high cell viability and osteogenic differentiation, and *in vivo* animal experiments further confirmed the ability of SU10 Mg to promote bone repair. Simultaneously, owing to the degradation of SA, the UFC carried by SUMg was released. Under near-infrared irradiation, the expected anti-tumor effects were observed. This offers a new strategy for repairing bone defects after surgery for OSCC.

## Data Availability

The original contributions presented in the study are included in the article/supplementary material, further inquiries can be directed to the corresponding author.
